# Effects of human‐induced prey depletion on large carnivores in protected areas: Lessons from modeling tiger populations in stylized spatial scenarios

**DOI:** 10.1002/ece3.5632

**Published:** 2019-09-08

**Authors:** Neil H. Carter, Simon A. Levin, Volker Grimm

**Affiliations:** ^1^ School for Environment and Sustainability University of Michigan Ann Arbor MI USA; ^2^ Department of Ecology & Evolutionary Biology Princeton University Princeton NJ USA; ^3^ Department of Ecological Modelling Helmholtz Centre for Environmental Research – UFZ Leipzig Germany

**Keywords:** agent‐based model, carnivore, conservation, prey depletion, protected areas, spatial heterogeneity, territoriality

## Abstract

Prey depletion is a major threat to the conservation of large carnivore species globally. However, at the policy‐relevant scale of protected areas, we know little about how the spatial distribution of prey depletion affects carnivore space use and population persistence. We developed a spatially explicit, agent‐based model to investigate the effects of different human‐induced prey depletion experiments on the globally endangered tiger (*Panthera tigris*) in isolated protected areas—a situation that prevails throughout the tiger's range. Specifically, we generated 120 experiments that varied the spatial extent and intensity of prey depletion across a stylized (circle) landscape (1,000 km^2^) and Nepal's Chitwan National Park (~1,239 km^2^). Experiments that created more spatially homogenous prey distributions (i.e., less prey removed per cell but over larger areas) resulted in larger tiger territories and smaller population sizes over time. Counterintuitively, we found that depleting prey along the edge of Chitwan National Park, while decreasing tiger numbers overall, also decreased female competition for those areas, leading to lower rates of female starvation. Overall our results suggest that subtle differences in the spatial distributions of prey densities created by various human activities, such as natural resource‐use patterns, urban growth and infrastructure development, or conservation spatial zoning might have unintended, detrimental effects on carnivore populations. Our model is a useful planning tool as it incorporates information on animal behavioral ecology, resource spatial distribution, and the drivers of change to those resources, such as human activities.

## Graphical abstract

We know little about how the spatial distribution of prey depletion affects tiger space use and population persistence. Using agent‐based simulation, we found that the spatial patchiness of prey resources, independent of total prey, strongly influences female tiger territory sizes, which in turn alters the carrying capacity of the landscape for tigers. Our model is a useful planning tool as it incorporates information on animal behavioral ecology, resource spatial distribution, and the drivers of change to those resources, such as human activities.

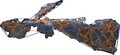

## 1 INTRODUCTION

Prey depletion is a major threat to the conservation of large carnivore species globally. One estimate indicates that over half of prey species for the clouded leopard (*Neofelis nebulosa*), Sunda clouded leopard (*Neofelis diardi*), tiger (*Panthera tigris*), dhole (*Cuon alpinus*), and the leopard (*Panthera pardus*) are classified as declining (Wolf & Ripple, 2016). Loss of prey species is caused by various human activities, such as overhunting, land‐use change, and competition with livestock (Ripple et al., 2015). Threats to prey species are widespread outside protected areas, since a vast majority of their populations occur in nonprotected forests where anthropogenic activities are more prevalent (Wolf & Ripple, 2016). However, prey depletion inside protected areas is also common, sometimes creating “empty forests” (Datta, Anand, & Naniwadekar, 2008). For example, in Bukit Barisan Selatan National Park on Sumatra, Indonesia, abundances of key prey species for tigers were 2–4 times higher in areas where the nearby human population density was lower, suggesting illegal hunting was depressing their numbers (O'Brien, Kinnaird, & Wibisono, 2003).

Human‐caused prey depletion varies across space. In India's Western Terai Arc Landscape, for example, densities of chital (*Axis axis*) and sambar (*Rusa unicolor*) were lower closer to the forest edge, where edge effects and livestock reduce forage (Harihar, Pandav, & Macmillan, 2014). Various studies have shown that the spatial distribution of prey affects the spatial distribution of their predators. The densities of ungulate prey, for instance, were key determinants of local tiger presence across a 38,000 km^2^ landscape in India (Karanth et al., 2011). Likewise, in Mozambique's Limpopo National Park, habitat use by African lions (*Panthera leo*) was most strongly influenced by the occurrence of their preferred prey (Everatt et al., 2015). Recent work also indicates that an animal will use smaller or larger areas depending on the patchiness of their habitats assuming the quality of the resources across the landscape is constant (Kie, Terry Bowyer, Nicholson, Boroski, & Loft, 2002; Mangipane et al., 2018). For mule deer (*Odocoileus hemionus*) in California, USA, for example, the spatial heterogeneity of habitats accounted for nearly 60% of the variability in the sizes of their home ranges (Kie et al., 2002). The spatial distribution of resources can therefore mediate the relationship between animal space use and fitness by increasing conspecific competition or likelihood of finding mates, which ultimately affects population dynamics (Carroll & Miquelle, 2006; Carroll, Phillips, Schumaker, & Smith, 2003).

**Figure 1:**
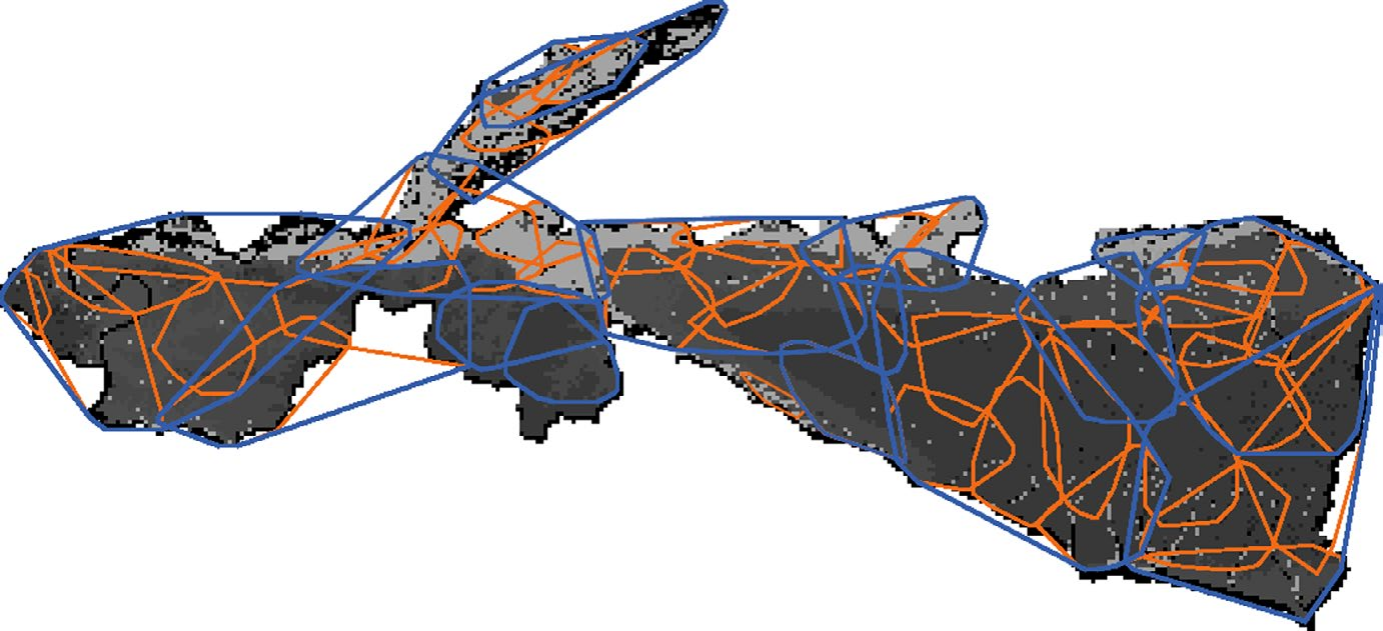
Snapshot of spatially explicit agent‐based model of tiger territory and population dynamics for Chitwan National Park, Nepal (see Carter et al., 2015 for details). Territories are outlined with 100% minimum convex polygons. Territories of females are orange and blue for males. Prey biomass production values ranged from a minimum of 2.05 kg per month per cell (dark gray) to 10.46 kg per month per cell (light gray), with a mean of 4.84 kg per month per cell

Although a number of studies have evaluated the extent to which the abundance of carnivores is coupled to the abundance of their prey, the effects of different spatial configurations of prey depletions on carnivore population dynamics have received much less attention. Yet, such information can inform conservation practice by elucidating the processes giving rise to spatiotemporal patterns of carnivore populations. Mechanistic computer models give us tools to evaluate future impacts of various prey depletion scenarios by more easily controlling for and experimentally manipulating sources of variability in the environment. Previous studies have used individual‐based approaches (Chapron et al., 2016; Imron, Herzog, & Berger, 2011; Watkins, Noble, Foster, Harmsen, & Doncaster, 2015) and perturbation analyses (Gosselin, Zedrosser, Swenson, & Pelletier, 2014; Lewis, Breck, Wilson, & Webb, 2014) to examine the effects of anthropogenic and natural factors on carnivore population dynamics. Although these studies have made important contributions, they did not explicitly evaluate the effects of spatially varying prey depletion. Therefore, we will expand on a previous agent‐based model of tiger territories and population dynamics (Carter, Levin, Barlow, & Grimm, 2015; Figure 1) to ask one key question: How do different spatial configurations of prey depletion affect tiger territory and population dynamics within an isolated protected area? We chose to focus on tigers for three main reasons. First, tigers are globally endangered (Joshi et al., 2016). Second, protected areas support critical source populations of tigers throughout their range; however, many of those areas face a great deal of human pressure, such as human‐induced prey depletion (Walston et al., 2010). Third, the spatial interactions between humans, ungulate prey species, and tigers are complex (Carter et al., 2013; Carter, Shrestha, Karki, Pradhan, & Liu, 2012; Harihar et al., 2014), necessitating tools for disentangling these interactions and informing tiger conservation within increasingly human‐dominated regions.

To address our research question, we approximate a continuum of prey depletion experiments in our computer model (see Section 2), ranging from experiments that constrain prey depletion close to the border of the protected area to those that allow prey depletion anywhere in the area. This continuum captures a number of ways that people have been observed encroaching on protected areas, for example, people overharvesting forage species (i.e., degrading prey habitat) along borders of protected areas or going deep into protected areas to hunt for bushmeat (Bouley, Poulos, Branco, & Carter, 2018; Carter et al., 2014). In addition to spatial extent, we control the intensity of prey depletion in our model, enabling us to make inferences on how different types of prey‐depleting human activities might affect tigers. For example, the presence of people may displace a proportion of prey from an area, whereas overhunting or land‐cover conversion might completely remove prey from an area. Our results will therefore provide insights for understanding and mitigating the effects of spatially varying prey depletion on the population dynamics of carnivores, like tigers. In addition to these stylized spatial scenarios, we also demonstrate the use of our model for real landscapes, by using the Chitwan National Park, Nepal, as an example.

## 2 MATERIALS AND METHODS

A detailed description of the model following the ODD protocol (Grimm et al., 2006, 2010) for describing agent‐based models and the NetLogo program that implemented the simulation experiments presented below are available online at https://github.com/nhcarter/tiger_abm. The model was parameterized for Chitwan National Park, where long‐term tiger behavioral and ecological data have been collected (Table 1). None of the model parameters were determined by fitting the full model to data, that is, by calibration; rather, most of them were measured directly from the field. A pattern‐oriented modeling approach (Grimm et al., 2005) was used to determine via visual inspection that a range of model outputs, such as reproduction rates, dispersal distances, resource selection, and land tenure, matched closely with observed patterns of the real tiger population in the park (Carter et al., 2015). Because of these strong correspondences, we are confident that the model performs well and would serve as an excellent foundation to address our central research question about the spatial heterogeneity of prey depletion.

**Table 1:** Summary of parameter information used in agent‐based model of tiger territory and population dynamics as they relate to spatially varying, prey depletion experiments

Parameters	Values	Reference	Notes
Age‐classes			
Breeding	3+ years old	Karanth and Stith (1999, p. 103)	Based on long‐term field data of tigers across sites.
Transient	2–3 years old		
Juvenile	1–2 years old		
Cub	0–1 years old		
Litter size distribution^a^			
1	0	Kenney et al. (2014, appendix A)	Based on long‐term field data of tigers in Chitwan.
2	0.23		
3	0.58		
4	0.17		
5	0.02		
Maximum number of cells female can add to territory per time step^a^	48 (3 km^2^)	Sunquist (1981, derived from table 15 on p. 37)	This value represents an approximation of the average area added to female's territory per month from observed data.
Annual survival^a^			
Breeding male	0.8	Karanth and Stith (1999, p. 103)	Survival rates were parameterized from field data on tigers, leopards, and cougars.
Breeding female	0.9		
Dispersal male	0.65		
Transient male	0.65		
Transient female	0.7		
Juvenile	0.9		
Cub	0.6		
Annual fecundity^a^			
Probability that 3‐year old resident female breeds	0.9	Kenney et al. (2014, appendix A)	Based on long‐term field data of tigers in Chitwan.
Probability that 4+ year old resident female breeds	1		
Maximum possible dispersal distance from natal range^a^			
Transient male	66 km	Smith (1993, table 1 p. 173)	Based on long‐term field data of tigers in Chitwan.
Transient female	33 km		
Prey thresholds^a^			
Minimum within territory	76 kg/month	Miller et al. (2014, p. 127)	Model estimates 2.5 kg/ day to maintain basal metabolic rate of female Bengal tiger in Bangladesh. This converts to: (2.5 kg/ day × 365 days)/12 months
Maximum within territory	167.3/month	Sunquist (1981, p 91)	From empirical data, estimates female tiger in Chitwan consumes 5–6 kg/ day. This converts to: (5.5 kg/day × 365 days)/12 months
Probability that dominant female will take territory patch from subordinate female if patch has highest prey^a^	0.25	Carter et al. (2015)	Based on expert opinion.
Proportion of prey within territory utilized by female tiger^a^	0.1	Karanth et al. (2004 (p. 4854)	Based on field data of large carnivore guilds across different sites in Asia and Africa.
Radius in which breeding males will search for nearby breeding females^a^	3 km	Ahearn et al. (2001, table 1 on p. 90)	Based on long‐term field data of tigers in Chitwan.
Max number of female territories a male can overlap^a^	6	Kenney et al. (2014, appendix A)	Based on long‐term field data of tigers in Chitwan.
Litter sex ratio at birth	50:50	Karanth and Stith (1999, p. 103)	Based on long‐term field data of tigers across sites.
Gestation period	3 or 4 months with equal probability	Sunquist et al. (1999, p. 7)	Gestation is 103 days, which is between 3 and 4 months. Model randomly selects either 3 or 4 months.
Search criteria for dispersing females to determine location of territory origin^a^			
Ideal area in which no other female territory occurs	12.57 km^2^ (2 km radius)		Based on expert opinion.
Less‐optimal area in which no other female territory occurs	3.14 km^2^ (1 km radius)	Carter et al. (2015)	
Probability that the transient male dies during challenge^a^	0.25	Kenney et al. (2014, appendix A)	Based on long‐term field data of tigers in Chitwan.
Probability that the breeding male dies during challenge^a^	0.6	Kenney et al. (2014, Appendix A)	Based on long‐term field data of tigers in Chitwan.
Probability offspring die due to infanticide following successful challenge^a^		Pusey and Packer (1994, derived from figure 1 on p. 279)	Based on long‐term field data on African lions in Tanzania's Serengeti National Park.
Juvenile	0.24		
Cub	0.79		

Note

The model was based on data collected largely in Nepal's Chitwan National Park.

a Parameters that were included in sensitivity analysis.

### 2.1 Landscapes

**Figure 2:**
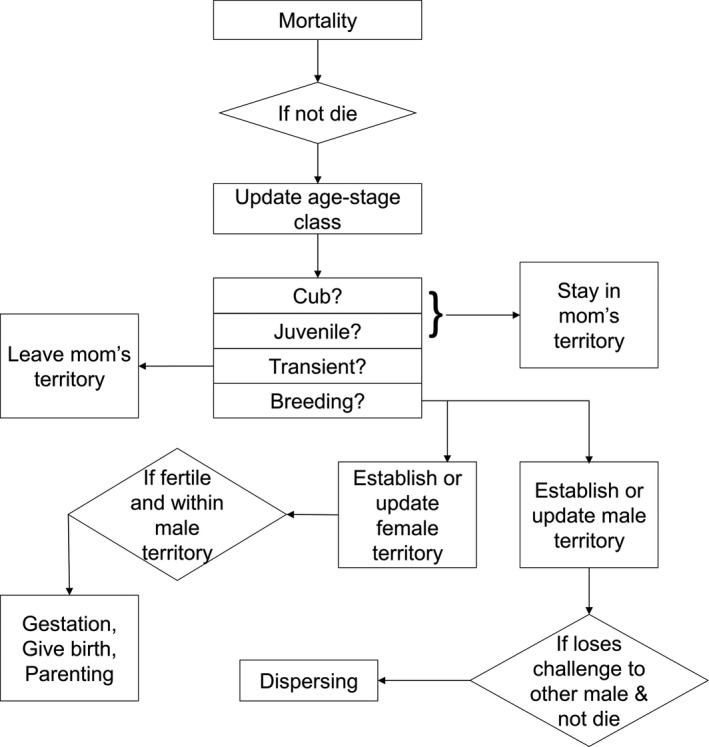
Overview of processes for spatially explicit, agent‐based model of tiger territory and population dynamics

The overall landscapes consist of 250 × 250 m cells. Each cell thus represents 62,500 m^2^ and was assigned a value for prey resources. We chose this cell size because it corresponds to the spatial resolution of prey data for the Nepal lowlands (Shrestha, 2004). We evaluated two model landscapes: one that is a stylized (circle) landscape with an area of 1,000 km^2^ and one that represents the real landscape of Chitwan National Park (~1,239 km^2^). The stylized landscape was chosen to develop and test the models of behavior and fine‐scale interactions of a large tiger population on a landscape with initially homogenous prey resources. We chose 1,000 km^2^ because it was equivalent to the size of many protected areas globally (Sanderson et al., 2006). In contrast, the Chitwan landscape was used to assess how prey depletions may affect tiger populations in a real landscape with complex spatial boundaries and highly heterogeneous prey resources. The distribution of prey resources in the Chitwan landscape was calculated by combining results from a Poisson regression relating land cover to prey abundances with empirical rates of average daily prey consumption by females and information on female territory sizes in Chitwan (Shrestha, 2004; Smith, McDougal, & Sunquist, 1987; Sunquist, 1981). Thus, the lower limit for prey biomass production in Chitwan was calculated as 2.05 kg per month per cell and the upper limit was 10.46 kg per month per cell, with an average of 4.84 kg per month per cell (Table 1, Figure 2). We used the average prey biomass production value in Chitwan (i.e., 4.84 kg/month) as the initial prey resource for each cell in the stylized landscape. Apart from our prey depletion experiments (see below), the prey resources in each cell were constant over time reflecting the simplifying model assumption that large‐scale seasonal shifts in prey biomass do not occur. In reality, tigers crop 10% of prey biomass per cell (Karanth, Nichols, Kumar, Link, & a, Hines JE., 2004), but we were ignoring this here as we are focusing on human‐induced changes in prey density, which often are larger and more permanent.

### 2.2 Tigers

Individual tigers are the agents in the model. In general, tigers in the model reproduce, disperse, establish and modify territories, and die, with other tigers dispersing to and establishing territories in the gaps left by dead tigers (Figure 2). Model behaviors reflect key behaviors documented in numerous ecological and behavioral studies of tigers (Ahearn, Smith, Joshi, & Ding, 2001; Imron et al., 2011; Karanth & Stith, 1999; Kenney, Allendorf, McDougal, & Smith, 2014; Miller et al., 2014; Smith, 1993; Sunquist, 1981; Sunquist, Karanth, & Sunquist, 1999). Tigers in the model have distinct behaviors associated with different age and stage classes, including cub, juvenile, transient, and breeding (Karanth & Stith, 1999). Cubs and juveniles stay in their natal territory (Figure 2). They die if their mom dies. Immediately following the juvenile stage, individuals enter the transient stage, which means that they are no longer associated with their natal range and therefore do not die if their mom dies. However, they do not have an explicit movement process until they reach the breeding stage. Upon reaching the breeding stage, females and males have different goals. Females form and defend territories that encompass a given amount of prey resources, whereas males form and defend territories that encompass a given number of females (Smith, 1993; Sunquist, 1981).

### 2.3 Territories

Although individual tigers are the agents in the model, their territories determine the spatial distribution of animals and regulate population dynamics over time (Seidensticker & McDougal, 1993; Smith et al., 1987). In addition, the territories are the link between the individual tigers and cell‐based prey resources. When females reach 3 years old, they disperse to a location where they establish the origin point of their territory. The territory origin point is determined by the female looking at all cells within a radius of 33 km of her natal origin (Smith, 1993) and selecting the cell with the highest mean prey resources and without another female within 2 km—sufficient space to establish a territory without immediate competition. However, if no cells meet that criteria (e.g., because of many females occupying the area), then she selects and moves to the cell that has the highest mean prey and no females within 1 km. If she cannot find a suitable location to establish her territory, then the female dies as the landscape has such a high density of other females that she would likely be unable to acquire enough food to survive. Once a female has established her territory starting point, she evaluates all neighboring cells adjoining her territory and adds the cell with the highest prey biomass production. She can add a total of 48 cells (3 km^2^) per time step (month), which is approximately the average area added to female's territory per month from observed data in Nepal (Sunquist, 1981). The cells of her territory cannot overlap those of another female territory. She continues to add cells to her territory until she acquires enough prey resources to maintain basal metabolic rate (76 kg/month, Carter et al., 2015). At which time, she can decide in each time step to drop cells in her territory in favor of adjacent cells with higher prey resources. This allows a female to adapt the size and shape of her territory to take advantage of the highest possible prey resources in the area. For example, if a breeding female dies and leaves her territory vacant as a result, then a neighboring female can adjust her territory to encompass portions of the deceased female's territory that have prey resources in excess of those in portions of her own territory (Smith et al., 1987). A dispersing female can establish her territory starting point in the deceased female's territory as well. That is, the territory of a deceased female can be split up in various ways among one or multiple other females. Furthermore, when two female territories come into contact with each other, each individual probabilistically decides to co‐opt cells from the other individual's territory, with the probability of winning based on age (Carter et al., 2015). When a female decides to challenge her neighbor and she wins, then she takes one cell from her opponent's territory rather than her whole territory. Therefore, female competition with other nearby females rarely leads to mortality, unless a female's territory is already so small to begin with that losing parts of her territory to other females could cause her to starve.

Males compete with other nearby males, and the outcomes of those challenges are more severe than for females. When males reach 3 years old, they move to acquire the nearest female within 66 km of their natal territory (Smith, 1993) that does not belong to another male (i.e., fall within another breeding male's territory). That is, when a male acquires a female, his territory encompasses her own. If no available female exists, the male will move to the closest female belonging to a male that he has not already challenged. The dispersing male will challenge the resident male for access to all his females. While searching for a territory, dispersing males will never challenge another male to whom they have already lost. This avoids dispersing males repeatedly challenging the same breeding male. The probability of the dispersing male winning is based on age (Kenney et al., 2014). If he wins, he gains access to all the females of his opponent. If he loses the challenge, there is a probability that he dies or simply moves on to another location to search for females or challenge males for access to females (Kenney et al., 2014). Breeding males that have lost the challenge and do not die will become dispersing males again (Figure 2). Males can overlap a maximum of six females (Kenney et al., 2014).

### 2.4 Experiments

**Figure 3:**
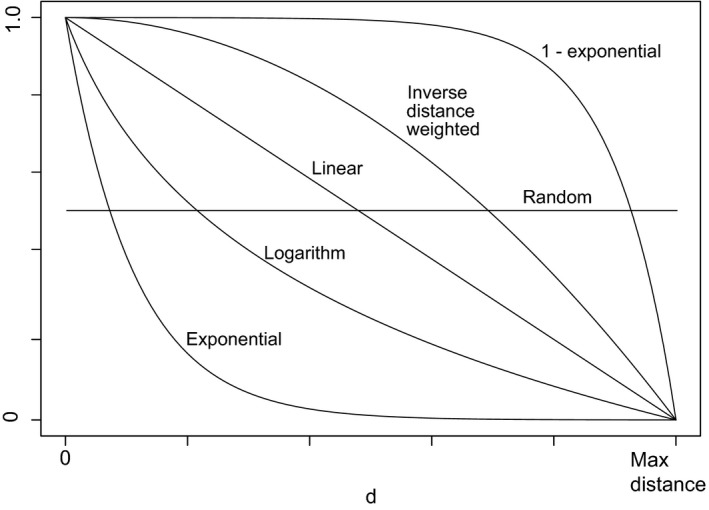
Five different mathematical functions that determine the probability of prey being depleted in a cell given the cell's distance (*d*) from the border of the interface between the protected area and human settlement. The spatial functions are exponential, logarithm, linear, inverse distance weighted, and the inverse of exponential, in order of increasing distance into the protected area that prey are more likely to be depleted. We also included a sixth experiment that randomly selected cells to deplete prey from, using a uniform distribution

**Figure 4:**
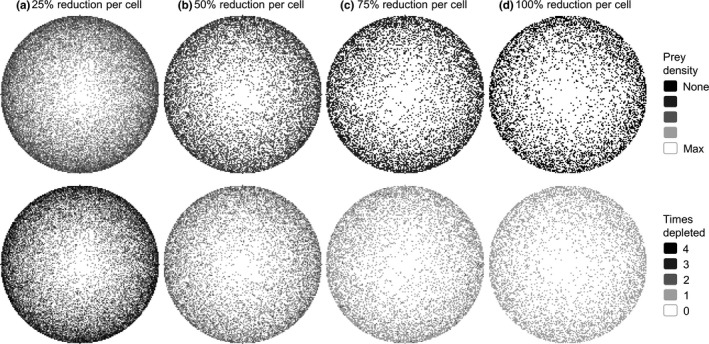
Top row shows prey density distribution in the stylized (circle) landscape following different levels of prey depletion per cell (25%, 50%, 75%, and 100%) as a function of distance from the border. Each simulated landscape has had 25% of the total prey depleted from the entire landscape using the *Logarithm* spatial function. Thus, although the cell‐level amount of prey varies, the total prey biomass is the same in each of the four simulated landscapes. Depending on the total prey biomass being removed from the landscape and the spatial function used, a cell may have its prey depleted multiple times. The images in the bottom row indicate the number of times a cell was probabilistically selected for depletion to reach the prey densities shown in the images above. These combinations of spatial functions, landscape depletion, and cell‐level depletion created landscapes with more or less spatial patchiness of prey resources for tigers

To represent a spatial continuum of prey depletion, we used six different mathematical functions that characterized the probability of prey depletion of cells at different distances from the border of the landscape (measured as nearest Euclidean distance). These spatial functions included exponential, logarithm, random, linear, inverse distance weighted, and the inverse of the exponential function (Figure 3). In addition, we used a combination of two methods to spatially vary the intensity of prey depletion. First, for each spatial function, we varied the overall depletion of prey across the whole landscape, ranging from depletions of 5%–25% of prey across the entire landscape at intervals of 5%. Once the landscape‐level depletion was set, we then varied the level of prey depletion that could occur per cell (i.e., 25%, 50%, 75%, or 100% prey depletion per cell). That is, we first set a target for total prey depletion across the landscape, for example, 5% depleted. The model implemented an algorithm that reached that target by varying how prey were depleted per cell, for example, 25% prey depleted per cell. Whether the prey in a cell was depleted by the set amount was based on the probabilities for each cell having its prey depleted assigned by the different spatial functions (i.e., lower probability with increasing distance from border). Each cell was selected randomly from all cells in the landscape without replacement. Once a cell was selected, a random number between 0 and 1 was drawn, and if that number was less than the probability of having its prey depleted based on the spatial function, then the prey was depleted by the per cell amount. After all cells had a chance to be selected, and their prey probabilistically depleted, the algorithm started again if the target amount of landscape‐level prey depletion was not reached. Therefore, a cell may have prey depleted more than once in order to reach the total prey depletion target (Figure 4). In those cases, the percent depleted per cell applied to the current prey density in the cell. By varying cell‐level prey depletion, landscape‐level prey depletion, and spatial function, we were able to assess how spatial heterogeneity of prey resources affects tiger territories and population dynamics in the landscape.

The models were initialized with 14 females and 7 males, numbers chosen to avoid large cycles in population size before activation of prey depletion experiments. The model runs for 200 time steps (months), approximately 5 tiger generations, without prey depletion to give the tiger population enough time to reach quasi‐stationary dynamics. Then, prey depletion scenarios occur instantaneously across the landscapes at time step 200. Although an instantaneous depletion of prey is unlikely, the range of cell‐level and landscape‐level values included in the model allow us to assess the effects of many different spatial configurations of prey depletion on tiger territories and populations.

### 2.5 Data analysis

Following the activation of prey depletion, the models were run for 480 time steps (40 years) because that was the maximum time it took for the simulations to reach a new quasi‐stationary state. Experiments were replicated 32 times, with the prey depletion algorithm rerun each time leading to different prey distributions, to take into account variation due to stochasticity included in the model. We chose 32 replications because the standard deviation in several model outputs appeared to stabilize after 30 replications. We did not conduct a sensitivity analysis here because we did so in Carter et al. (2015). In that sensitivity analysis, we varied fourteen input parameters (Table 1) from their reference value and compared outputs for tiger population size, total breeding animals, and female territory size over time (Carter et al., 2015). For all parameters except one (breeding female annual survival rate), the changes in model outputs were proportionally similar to or less than the changes to input parameter values.

We calculated Moran's *I*, a coefficient of autocorrelation, to measure the spatial heterogeneity of prey resources for each experiment. Values of Moran's *I* near zero indicate randomness, whereas values near 1 indicate high spatial correlation in prey. We used R packages, RNetLogo, and spdep (Bivand et al., 2019; Thiele, Kurth, & Grimm, 2012), to calculate the global Moran's *I* for the 120 distinct experiments; that is, a single Moran's *I* value was applied to all 32 replicates of each experiment. We also calculated the tiger population size and mean female territory size for the last time step of each of the 32 replicates of each of the 120 experiments (6 spatial functions × 5 landscape‐level depletion scenarios × 4 cell‐level depletion scenarios). This produced 3,840 values for each of the two outcome variables. Finally, we calculated these outcome variables for 32 replicates of the control scenario, where there was no prey depletion, to compare against the experiments. We focused on female territory size instead of male territory size, because female territories are determined by the spatial distribution of prey resources, whereas males align themselves with the females.

Next, we fitted generalized linear models to Moran's *I*, female territory size, and total tiger population size using our three prey depletion factors—spatial function, landscape depletion, and cell depletion—as fixed effects. For Moran's *I* and female territory size, we fitted the models using a gamma distribution with a log‐link. For total tiger population size, we fitted the models using a negative binomial distribution with a log‐link. We also fitted generalized linear models to female territory size and total tiger population size that only included Moran's *I* values while controlling for landscape depletion.

Lastly, we examined whether differences in the prey distributions between the stylized and real landscapes altered the rates of female starvation. We chose female starvation because it is tied directly to female competition for prey resources and because females are a primary driver of population change. For both the stylized and real landscapes, we first calculated the average number of females that died from starvation per month over the last 100 months of each model run (i.e., 32 replications for each experiment and control). Using those estimates, we plotted trends in female starvation as a linear function of landscape depletion for each level of cell depletion. We then calculated the overall rate of female starvation for each spatial function and visually compared those values to the control (i.e., no prey depletion). We also visually compared rates between the stylized and real landscapes. All analyses were performed using the R software (R Core Team, 2019).

## 3 RESULTS

### 3.1 Spatial heterogeneity of prey resources

Values of Moran's *I*, which we used as our measure of spatial autocorrelation, ranged from 0.41 to 0.98 in the stylized (circle) landscape and 0.21 to 0.71 in the real (Chitwan) landscape. Moran's *I* was strongly influenced by all prey depletion factors, with greater cell‐level and landscape‐level prey depletion values producing lower Moran's *I* values (i.e., less spatially homogeneous prey) in both the stylized and Chitwan landscapes (Table 2). The cell‐level depletion values of 75% and 100% had large negative effects on Moran's *I*, indicating that high levels of fine‐scale depletion created patchier resource environments in our simulations. The *Exponential* spatial function produced higher Moran's *I* (i.e., more spatially autocorrelated) than the other spatial functions (Table 2). This is because the *Exponential* spatial function only selects cells close to the edge, which can be depleted several times, creating more homogenous prey distributions than other spatial functions that create patchier environments by depleting cells farther into the protected area.

**Table 2:** Generalized linear models (gamma distribution, log‐link) for Moran's/in the simulated stylized and real landscapes

Factor	Stylized (circle) landscape	Real (Chitwan) landscape
	Estimate (90% CI)	Estimate (90% CI)
Cell 25%	−0.02 (−0.05, 0.01)	0.05 (0.02, 0.09)^a^
Cell 50%	−0.15 (−0.18, −0.12)^a^	−0.08 (−0.11, −0.05)^a^
Cell 75%	−0.28 (−0.31, −0.25)^a^	−0.22 (−0.25, −0.19)^a^
Cell 100%	−0.39 (−0.42, −0.36)^a^	−0.33 (−0.36, −0.3)^a^
Landscape 10%	−0.09 (−0.1, −0.08)^a^	−0.08 (−0.09, −0.07)^a^
Landscape 15%	−0.16 (−0.17, −0.15)^a^	−0.15 (−0.16, −0.14)^a^
Landscape 20%	−0.22 (−0.23, −0.21)^a^	−0.21 (−0.22, −0.2)^a^
Landscape 25%	−0.27 (−0.28, −0.26)^a^	−0.26 (−0.27, −0.25)^a^
Exponential	0.33 (0.32, 0.34)^a^	0.42 (0.41, 0.43)^a^
Logarithm	0.06 (0.06, 0.07)^a^	−0.03 (−0.04, −0.02)^a^
Linear	0.04 (0.03, 0.05)^a^	−0.03 (−0.04, −0.02)^a^
Inverse distance weighted	0.02 (0.01, 0.03)^a^	−0.02 (−0.03, −0.01)^a^
1‐Exponential	0.02 (0.01, 0.03)^a^	0.01 (0, 0.02)^a^

Note

Three prey depletion factors—spatial function, landscape depletion, and cell depletion—were included as covariates. Reference categories for each factor included the control settings where no prey were depleted. Landscape depletion 5% and the random spatial function categories were removed because they were unidentifiable (linearly dependent) predictor variables.

a The factor has 90% confidence intervals that do not cross zero.

### 3.2 Determinants of female territory size

**Figure 5:**
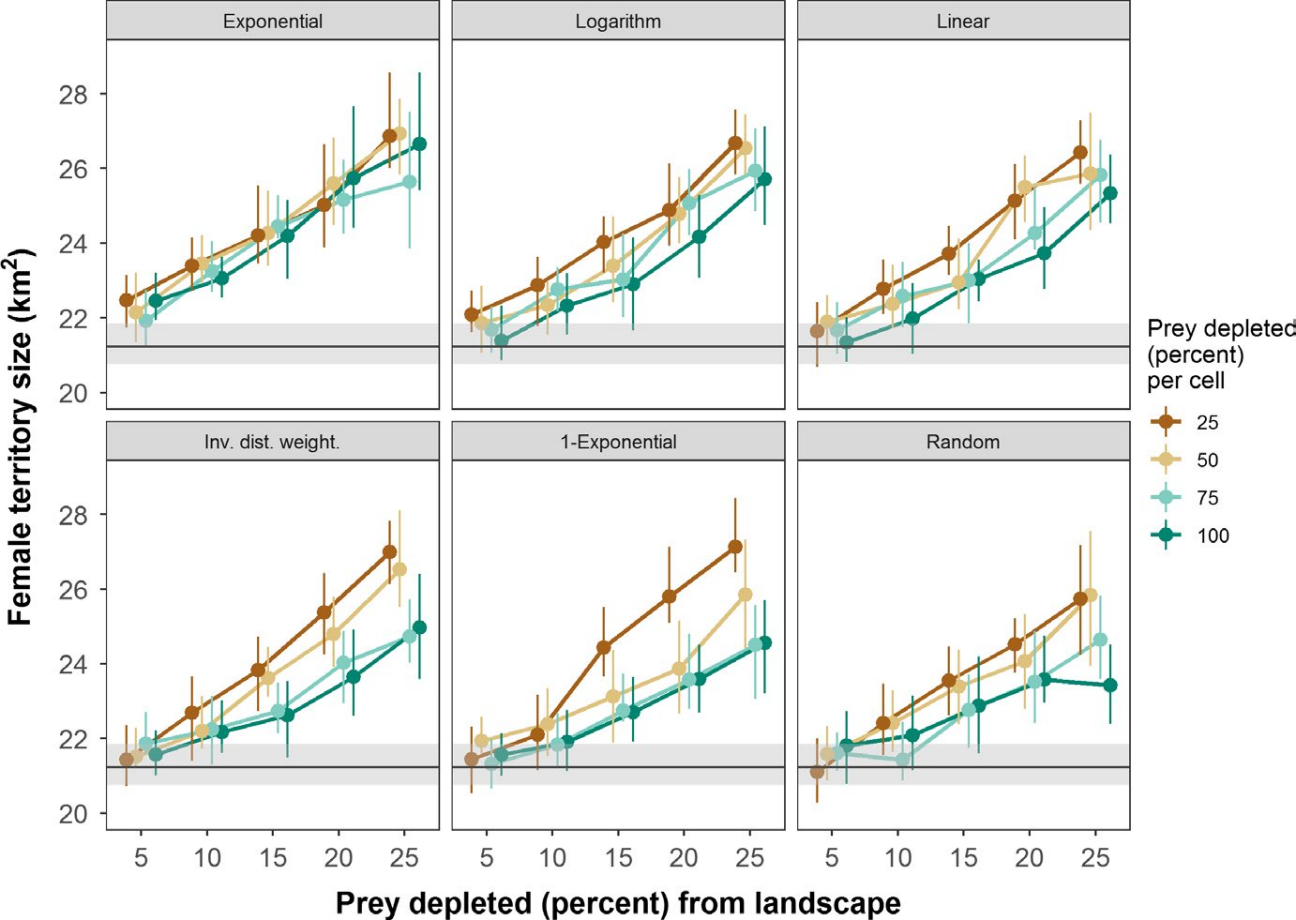
Female territory sizes in the real (Chitwan) landscape for different prey depletion experiments. The size was calculated from the last time step of the simulation and averaged over 32 replicates. The 25% and 75% quartiles around the mean are indicated by vertical lines. The results have been horizontally staggered from each other so they can be more easily distinguished. The mean female territory size of the control (i.e., no prey depletion) is shown as horizontal, black lines with 25% and 75% quartiles shown as gray ribbons

**Figure 6:**
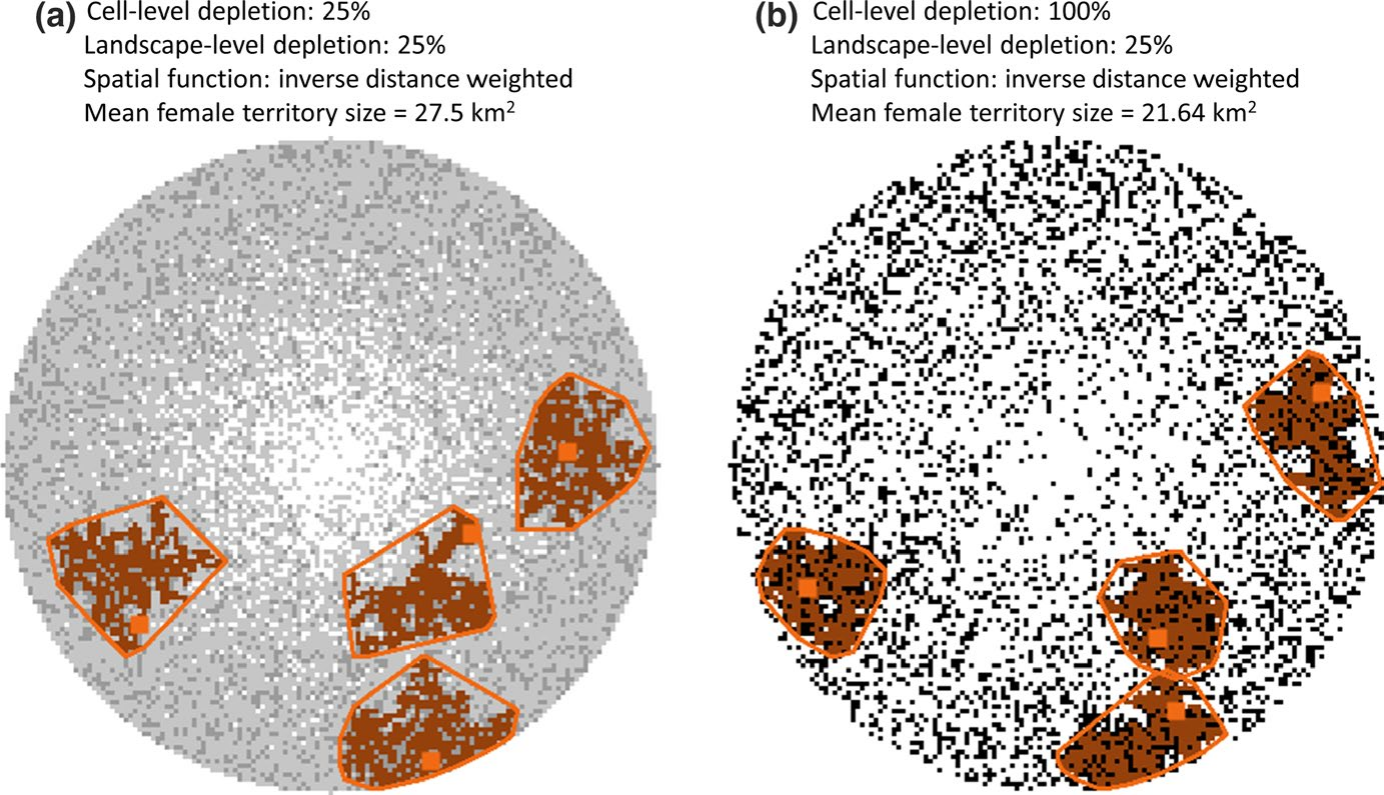
Comparison of female territory sizes (orange) in two stylized experiments. Females were initialized in the exact same locations prior to growing territories. Prey resources per cell range from low (black) to high (white). Both landscapes have the same total amount of prey depleted from the landscape (25%), and the same spatial function was used (Inverse Distance Weighted) for determining where to deplete prey, but (a) used cell‐level depletion of 25% to reach the total prey depletion target, whereas (b) used cell‐level depletion of 100%. Thus, the spatial distribution of prey resources was less patchy in (a) than (b). Mean female territory size was 27% larger in (a) than (b)

Across all experiments, female territory size ranged from 17.5 to 29.77 km^2^ in the stylized (circle) landscape and 15.68 to 30.44 km^2^ in the real (Chitwan) landscape. Landscape‐level depletion of prey, among the three factors, had the strongest influence on female territory size in both landscapes (Table 3, Figure 5). When accounting for landscape‐level prey depletion, experiments that removed 25% of prey per cell positively influenced female territory size (Table 3, Figure 6). Exponentiating the coefficients in Table 3 indicated that the cell‐level depletion of 25%, alone, increased the mean female territory size by 4% compared to the control in the stylized landscape (20.51–21.29 km^2^) and over 2% in the Chitwan landscape (21.23–21.69 km^2^). In the stylized landscape, only the *Logarithm* spatial function, in which prey depletion extends roughly halfway into the simulated landscape, had a positive effect on female territory size (Table 3). In contrast, in the real landscape, all of the spatial functions had a positive effect on female territory size. The size of the effect corresponded to the degree to which depletion occurred close to the protected area border, such that the *Exponential* function (i.e., depletion closest to border) had the largest positive effect and the *1‐Exponential* function (i.e., depletion farthest from border) had the smallest positive effect (Table 3). The *Exponential* function, alone, increased the mean female territory size by over 5% compared to the control. Finally, when accounting for landscape‐level prey depletion, Moran's *I* was positively related to female territory size (Table 4). That is, more spatially homogeneous prey resources were related to larger female territories.

**Table 3:** Generalized linear models (gamma distribution, log‐link) for female territory size in the simulated stylized and real landscapes

Factor	Stylized (circle) landscape	Real (Chitwan) landscape
	Estimate (90% CI)	Estimate (90% CI)
Cell 25%	0.04 (0.02, 0.05)^a^	0.02 (0, 0.04)^a^
Cell 50%	0.02 (0, 0.03)^a^	0.01 (−0.01, 0.03)
Cell 75%	0 (−0.01, 0.02)	−0.01 (−0.03, 0.01)
Cell 100%	−0.01 (−0.02, 0.01)	−0.01 (−0.03, 0.01)
Landscape 10%	0.03 (0.03, 0.04)^a^	0.03 (0.03, 0.04)^a^
Landscape 15%	0.07 (0.06, 0.07)^a^	0.07 (0.07, 0.08)^a^
Landscape 20%	0.11 (0.1, 0.11)^a^	0.12 (0.12, 0.13)^a^
Landscape 25%	0.15 (0.14, 0.15)^a^	0.17 (0.17, 0.18)^a^
Exponential	0.01 (0, 0.01)^a^	0.05 (0.05, 0.06)^a^
Logarithm	0.01 (0.01, 0.01)^a^	0.02 (0.02, 0.03)^a^
Linear	0.01 (0, 0.01)^a^	0.02 (0.01, 0.02)^a^
Inverse distance weighted	0 (0, 0.01)	0.01 (0.01, 0.02)^a^
1‐Exponential	0 (0, 0.01)	0.01 (0, 0.01)^a^

Note

Three prey depletion factors—spatial function, landscape depletion, and cell depletion—were included as covariates. Reference categories for each factor included the control settings where no prey were depleted. Landscape depletion 5% and the random spatial function categories were removed because they were unidentifiable (linearly dependent) predictor variables.

a The factor has 90% confidence intervals that do not cross zero.

**Table 4:** Generalized linear models (gamma distribution, log‐link) for female territory size in the simulated stylized and real landscapes

Factor	Stylized (circle) landscape	Real (Chitwan) landscape
	Estimate (90% CI)	Estimate (90% CI)
Moran's I	0.12 (0.11, 0.13)^a^	0.2 (0.18, 0.21)^a^
Landscape 5%	0.03 (0.02, 0.05)^a^	0.03 (0.01, 0.05)^a^
Landscape 10%	0.07 (0.06, 0.09)^a^	0.07 (0.05, 0.09)^a^
Landscape 15%	0.11 (0.1, 0.13)^a^	0.12 (0.1, 0.14)^a^
Landscape 20%	0.16 (0.14, 0.18)^a^	0.17 (0.15, 0.19)^a^
Landscape 25%	0.2 (0.19, 0.22)^a^	0.22 (0.2, 0.24)^a^

Note

Moran's *I* and landscape‐level depletion (percentages) included as covariates. Landscape depletion scenario where no prey removed (control) used as reference category.

a The factor has 90% confidence intervals that do not cross zero.

### 3.3 Determinants of tiger population size

**Figure 7:**
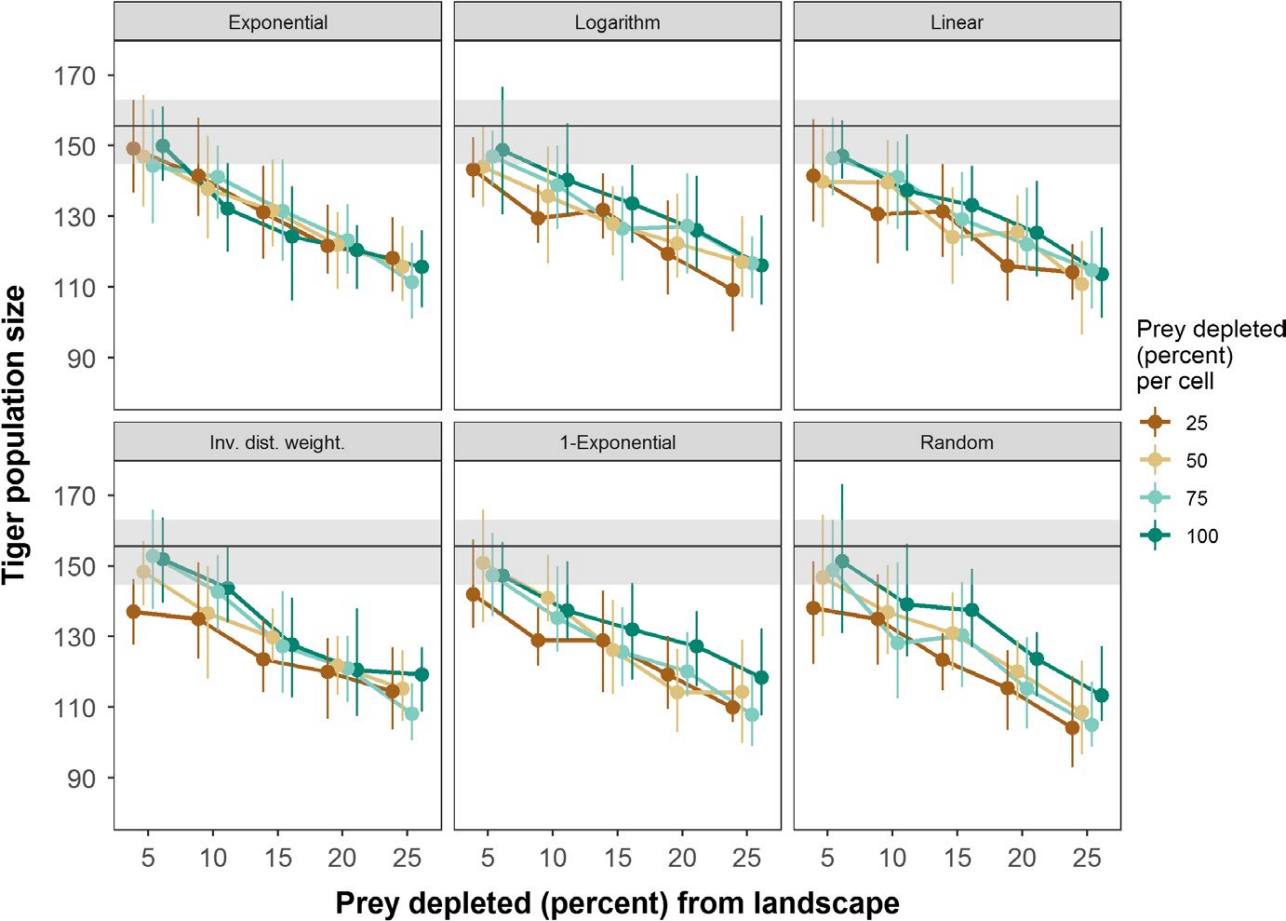
Tiger population sizes in the real (Chitwan) landscape for different prey depletion experiments. The size was calculated from the last time step of the simulation and averaged over 32 replicates. The 25% and 75% quartiles around the mean are indicated by vertical lines. The results have been horizontally staggered from each other so they can be more easily distinguished. The mean tiger population size of the control (i.e., no prey depletion) is shown as horizontal, black lines with 25% and 75% quartiles shown as gray ribbons

**Figure 8:**
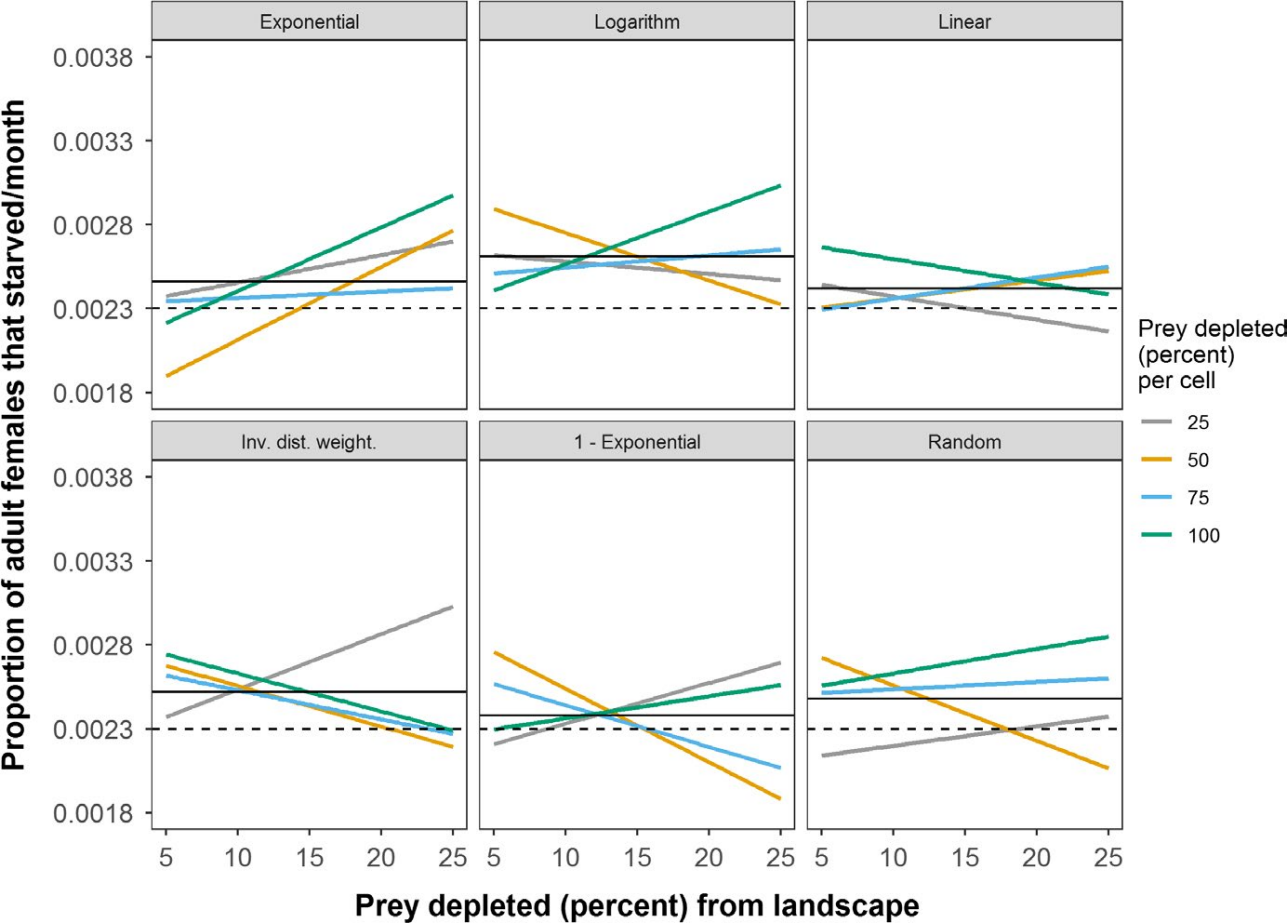
Female tiger starvation rates in the stylized (circle) landscape for different prey depletion experiments. Rates are calculated as proportion of adult females that starved per time step (month), averaged for the last 100 time steps of each simulation, averaged across the 32 replicates of each experiment, grouped for each spatial function, and regressed against levels of landscape depletion of prey. The mean female tiger starvation rate of the control (i.e., no prey depletion) is shown as horizontal, gray dashed lines. Horizontal black lines indicate the mean for each respective set of experiments

**Figure 9:**
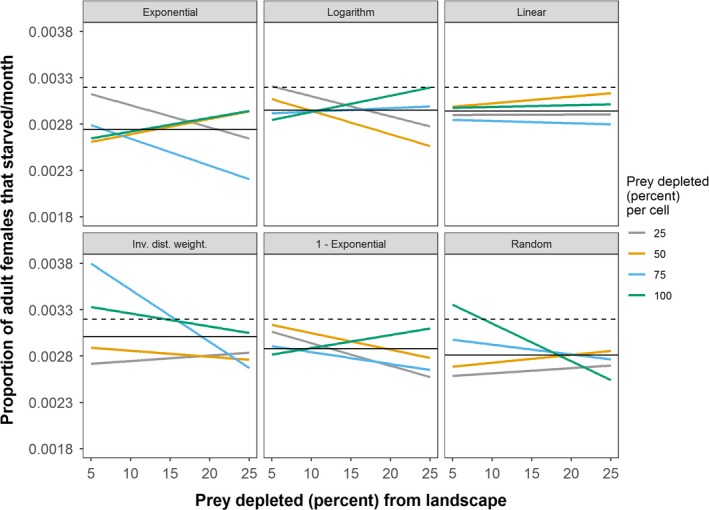
Female tiger starvation rates in the real (Chitwan) landscape for different prey depletion experiments. Rates are calculated as proportion of adult females that starved per time step (month), averaged for the last 100 time steps of each simulation, averaged across the 32 replicates of each experiment, grouped for each spatial function, and regressed against levels of landscape depletion of prey. The mean female tiger starvation rate of the control (i.e., no prey depletion) is shown as horizontal, gray dashed lines. Horizontal black lines indicate the mean for each respective set of experiments

Across all experiments, tiger population size ranged from 25 to 202 individuals in the stylized (circle) landscape and 38 to 225 individuals in the real (Chitwan) landscape. Landscape‐level depletion of prey, among the three factors, had the strongest influence on tiger population sizes (Table 5, Figure 7). When accounting for landscape‐level depletion, cell‐level prey depletion negatively influenced tiger population size, with cell‐level depletion of 25% having the strongest negative effect (Table 5). Exponentiating the coefficients in Table 5 indicated that cell‐level depletion of 25%, alone, decreased the mean tiger population size by 13% compared to the control in the stylized landscape (156 to 136 individuals) and over 9% in the real (Chitwan) landscape (156 to 141 individuals). Counterintuitively, the *Exponential*, *Logarithm*, and *Inverse Distance Weighted* spatial functions had positive effects on tiger population size in the real landscape, not in the stylized landscape (Table 5). In the stylized landscape, female starvation rates were generally higher for all spatial functions than in the control (no prey depletion) simulations (Figure 8). Whereas, in the real landscape, female starvation rates were generally lower for all spatial functions than the control (Figure 9). In the real landscape, for example, the rate of female starvation was 16.7% lower in experiments that used the *Exponential* function (0.00274 proportion of adult females/month) than in the control scenario (0.00319 proportion of adult females/month, Figure 9). This suggests that the unique distribution of prey resources in Chitwan mediated the effects on tiger populations of prey depletion that varies in space (see Section 4). Finally, when accounting for landscape‐level prey depletion, Moran's *I* was negatively related to tiger population sizes (Table 6). That is, more spatially homogeneous prey resources were related to smaller tiger population sizes.

**Table 5:** Generalized linear models (negative binomial distribution, log‐link) for tiger population size in the simulated stylized and real landscapes

Factor	Stylized (circle) landscape	Real (Chitwan) landscape
	Estimate (90% CI)	Estimate (90% CI)
Cell 25%	−0.14 (−0.18, −0.09)^a^	−0.1 (−0.14, −0.05)^a^
Cell 50%	−0.12 (−0.16, −0.07)^a^	−0.08 (−0.12, −0.03)^a^
Cell 75%	−0.09 (−0.13, −0.04)^a^	−0.08 (−0.12, −0.03)^a^
Cell 100%	−0.07 (−0.12, −0.03)^a^	−0.06 (−0.1, −0.01)^a^
Landscape 10%	−0.08 (−0.09, −0.07)^a^	−0.07 (−0.08, −0.05)^a^
Landscape 15%	−0.14 (−0.15, −0.12)^a^	−0.12 (−0.14, −0.11)^a^
Landscape 20%	−0.21 (−0.23, −0.2)^a^	−0.19 (−0.2, −0.18)^a^
Landscape 25%	−0.31 (−0.32, −0.29)^a^	−0.26 (−0.27, −0.25)^a^
Exponential	0 (−0.02, 0.01)	0.02 (0.01, 0.04)^a^
Logarithm	0 (−0.01, 0.02)	0.02 (0.01, 0.03)^a^
Linear	−0.01 (−0.02, 0.01)	0.01 (0, 0.03)^a^
Inverse distance weighted	−0.01 (−0.02, 0.01)	0.02 (0, 0.03)^a^
1‐Exponential	−0.01 (−0.02, 0.01)	0.01 (0, 0.02)

Note

Three prey depletion factors—spatial function, landscape depletion, and cell depletion—were included as covariates. Reference categories for each factor included the control settings where no prey were depleted. Landscape depletion 5% and the random spatial function categories were removed because they were unidentifiable (linearly dependent) predictor variables.

a The factor has 90% confidence intervals that do not cross zero.

**Table 6:** Generalized linear models (negative binomial distribution, log‐link) for tiger population size in the simulated stylized and real landscapes

Factor	Stylized (circle) landscape	Real (Chitwan) landscape
	Estimate (90% CI)	Estimate (90% CI)
Moran's I	−0.16 (−0.19, −0.13)^a^	−0.04 (−0.08, 0)^a^
Landscape 5%	−0.13 (−0.17, −0.08)^a^	−0.06 (−0.11, −0.02)^a^
Landscape 10%	−0.22 (−0.26, −0.17)^a^	−0.13 (−0.17, −0.09)^a^
Landscape 15%	−0.28 (−0.33, −0.24)^a^	−0.19 (−0.23, −0.15)^a^
Landscape 20%	−0.37 (−0.41, −0.32)^a^	−0.25 (−0.3, −0.21)^a^
Landscape 25%	−0.47 (−0.51, −0.42)^a^	−0.33 (−0.37, −0.28)^a^

Note

Moran's *I* and landscape‐level depletion (percentages) included as covariates. Landscape depletion scenario where no prey removed (control) used as reference category.

a The factor has 90% confidence intervals that do not cross zero.

## 4 DISCUSSION

Although prey depletion is a major threat to carnivore populations around the world, very few studies examine how different spatial configurations of human‐induced prey depletion affect carnivore population dynamics. We developed a computer simulation to experimentally test how a spatial continuum of prey depletion scenarios affects tiger populations in protected areas. We found that the spatial patchiness of prey resources influences female tiger territory sizes, which in turn alters the carrying capacity of the landscape for tigers. We also found that density‐dependent, female mortality due to resource deprivation (i.e., starvation) mediates the effects of spatially heterogeneous prey depletion on tiger populations.

For a given level of prey depletion at a landscape scape, we found that simulations with more spatially homogeneous prey tended to have smaller tiger population sizes over time than simulations with less spatially homogeneous prey resources. This outcome is caused by the spatial distribution of prey resources modifying tiger territory dynamics. Female tiger territory sizes grew larger as prey resources became more spatially homogeneous (i.e., spread out more evenly), suggesting that individuals reach their energetic requirements in smaller areas in landscapes characterized by patchier distributions of resources when total prey resources in the landscape were held constant. This might be explained by the observation that experiments that partially depleted prey over a greater proportion of the protected area created more spatially autocorrelated distributions of prey, such that relatively large areas become less suitable as habitat for tigers. In contrast, experiments that completely depleted prey from spatially localized areas (i.e., cell‐level depletion of 100%) generated more spatially dissimilar distributions of prey, such that relatively small areas would have little‐to‐no prey while other larger areas would have very high prey resources, that is, more suitable habitat. As a consequence, territories were larger for tigers in experiments with spatially homogeneous, partially depleted prey resources because individuals would have to range farther to encompass a sufficiently large area, whereas the locally high levels of remaining prey resources in experiments with prey depleted more heterogeneously enable individuals to have smaller territories (Figure 6).

Where prey was depleted, based on the continuum of spatial functions, also influenced spatial heterogeneity of prey resources and female territory sizes. The *Exponential* function, for example, concentrated prey depletion along the boundaries of the landscape, which effectively shrinks the area of the landscape with available prey resources. Given that the highest prey densities in Chitwan occur in the riverine forest and grasslands along the northern border (Carter et al., 2013; Figure 1), the *Exponential* function affected the portions of the park with the most abundant prey resources and thereby disproportionately increased female territory sizes. Because territories determine how a population of animals distribute themselves across a landscape, larger territories mean that a landscape can support fewer tigers than if those animals had smaller territories.

Territorial predators, like tigers, can compete intensely against one another for areas holding high numbers of prey. In the Chitwan landscape, depleting prey along the edge of the park (e.g., when using the *Exponential* function), while increasing female territory sizes and decreasing tiger numbers overall, also decreased female competition for those areas, leading to lower rates of female starvation (Figure 9). Empirical evidence indicates that human presence is high along the border of the park, potentially decreasing tiger habitat quality over time (Carter et al., 2013, 2012). It is unclear whether competition‐induced tiger mortality has changed over time as a result. If, unlike Chitwan, prey densities were higher farther inside a protected area, then prey depletion along the border might exacerbate agonistic interactions in the interior of the park and increase mortality rates. Likewise, restoring prey in high numbers in certain areas might allow tiger numbers to grow but could also increase tiger mortality due to heightened competition for those prey resources. Our findings therefore underscore the importance of understanding fine‐scale prey distributions across areas that support predators, as they can influence the impact of human‐induced prey depletion and conservation interventions on predator numbers (Jackson & Fahrig, 2016).

We implicitly incorporated a cost to larger territories via a cap in home range size. Alternatively, one could explicitly incorporate these costs, for example, by having tigers expend more calories when ranging farther to defend a larger territory (Shepard et al., 2013). Inclusion of these costs might alter model outcomes because animals may energetically be unable to encompass enough resources to survive and, as a consequence, die. However, these travel costs may be relatively low for tigers in prey‐rich environments, such as the Chitwan landscape in Nepal that we based our model on. Where prey biomass is high, tigers can encompass sufficient prey within smaller areas compared to tigers in other parts of their range (Simcharoen et al., 2014). In those cases, increased mortality due to energetic costs of a slightly larger territory is unlikely. In contrast, in places where tigers have very large territories and prey biomass is low, such as in the Russian Far East, the energetic costs associated with increasing territory sizes could substantially increase tiger mortality rates (Carbone et al., 2002). Future work including energetic costs of movement and prey‐poor landscapes is needed.

We did not incorporate into our model dynamic changes in prey resources, for example, as a function of season, hunting pressure, or demography. Although doing so could better reflect the complexity of predator–prey interactions in space and time, it would require parameterizing relationships (e.g., interactive effects of predation levels, forage quality, and intraspecific competition on prey abundance within and across cells) for which we have scant or no data and was thus considered beyond the scope of this preliminary analysis. Here, we wanted to focus on how the simple redistribution of prey resources, independent of total prey in the landscape, can influence tiger populations. It is important, however, to validate simulation outcomes with field evidence, such as through pattern matching and tools including Approximate Bayesian Computation (Beaumont, 2010; Kang & Aldstadt, 2018). To do so would require more empirical data on tiger movement, territory size, and population fluctuation in response to human‐induced changes to the spatial distributions of prey. For example, field data on animal abundance and movement (e.g., from camera traps or GPS collars) combined with remotely sensed data (e.g., land‐cover structure and composition) could be used to empirically relate carnivore territory size to varying levels of spatial patchiness of prey resources, and help determine the role that the spatial heterogeneity in food has in distributing carnivores. If conducted across sites with a gradient a human disturbance, such analyses could help establish a clear link among human activities, spatial distributions of prey, and carnivore population dynamics.

Nevertheless, our results might help explain patterns of animal space use in human‐modified landscapes. In particular, our findings suggest that an animal will adjust, or expand, its territory to meet energetic requirements if it is getting less resources from some portions of its territory in the presence of people. Diminished access to resources can either be caused by direct removal of food resources by people or fear of people. This appears to be the case for moose (*Alces alces*), which increased their home range sizes when disturbed by humans (Andersen, Linnell, & Langvatn, 1996). Likewise, the territory of coyotes (*Canis latrans*) was larger in Tucson, a highly human‐modified area, than has been found by most researchers (Grinder & Krausman, 2001). Tiger territories in India's Panna Tiger Reserve, where anthropogenic disturbance is high, were between three and four times larger than territories reported for other tropical habitats with comparable prey densities (Chundawat, Sharma, Gogate, Malik, & Vanak, 2016). Indeed, specific prey depletion experiments could help predict the altered space use of animals in protected areas due to the spatial distribution of human activities (Bino et al., 2010). For example, the *Exponential* spatial function, concentrating resource depletion along the border of a protected area, might be able to recreate the pattern of lions moving farther away from the edge of the Masai Mara National Reserve, Kenya, as human activity increased along the border (Green, Johnson‐Ulrich, Couraud, & Holekamp, 2017). Likewise, the *Logarithm* spatial function, concentrating resource depletion farther into animal habitats, could be used to assess the population consequences of Eurasian lynx (*Lynx lynx*) adjusting their home ranges to minimize their exposure to high levels of spatially dispersed human disturbance in Norway (Bouyer et al., 2015). By explicitly accounting for spatial distributions of resources, our model can better account for the effects of various human activities on animal space use and population dynamics than standard foraging models that only include overall resource productivity.

Our results have implications for how we evaluate animal conservation strategies on anthropogenic landscapes. Much of the remaining ranges for many carnivore species are outside protected area networks in multiuse areas where human activities are pervasive (Chapron et al., 2014; Swanepoel, Lindsey, Somers, Hoven, & Dalerum, 2013; de la Torre, González‐Maya, Zarza, Ceballos, & Medellen, 2017). We would expect based on our model results that territorial carnivore species will have different space‐use patterns outside protected areas compared to inside them, given the effects of human activities on distributions of prey (Baeza & Estades, 2010). Human activities in multiuse areas (e.g., collection of forest products) may reduce the capacity for carnivores to exploit prey resources over large spatial areas, subsequently causing these animals to adjust, or enlarge, their home ranges. Such cases are relevant to current discussions about the merits of a land‐sharing approach for carnivore conservation (Crespin & Simonetti, 2018; López‐Bao, Bruskotter, & Chapron, 2017), in which human land‐uses and wildlife habitats spatially overlap, as opposed to a land‐sparing approach, in which human land‐uses are spatially separated from core wildlife habitats (Luskin, Lee, Edwards, Gibson, & Potts, 2018). On one hand, in addition to supporting fewer carnivores, larger territories in shared landscapes could potentially increase the frequency of edge effects (e.g., anthropogenic mortality) and depredation of livestock (Crespin & Simonetti, 2018), threatening recovery efforts for carnivore species. On the other hand, if anthropogenic mortality was minimized, land‐sharing would provide a greater area over which carnivores could distribute themselves than in those lands currently under protection (López‐Bao et al., 2017), increasing the likelihood of population persistence. To date, very little attention has been paid to understanding how the spatially varying interspersion of human disturbances in shared landscapes alters carnivore behavior, space use, and population dynamics.

There is an urgent need for spatial planning tools that incorporate information on animal behavioral ecology, resource spatial distribution, and the drivers of change to those resources, such as human activities. Our model is a prototype of one such planning tool. When combined with and validated by empirical observations, the model can help predict how a range of conservation strategies and development policies across different land management regimes will affect carnivore population dynamics by altering the spatial configuration of prey resources. These predictive capacities would be useful for evaluating, for instance, how animals will distribute themselves and utilize resources in community forests or conservancies adjacent to protected areas, or how recreational activities, such as hiking or mountain biking, displace or influence predator–prey systems in public lands (Gaynor, Hojnowski, Carter, & Brashares, 2018; Rodríguez‐Prieto et al., 2014). Managers could use a model like ours to evaluate how ungulate harvest levels in different game management units might influence predator population size. It can also help guide decision‐making on where to locate roads and railways by assessing the potential long‐lasting effects of linear infrastructure on wildlife dynamics in spatially complex landscapes (Torres, Jaeger, & Alonso, 2016). Moreover, our model can help with large landscape planning, such as evaluating alternative locations for refuges, impacts of degazzetting parks, and assessing whether current networks of nature reserves allow animal adaptation to climate change (Mascia & Pailler, 2011; Mawdsley, O'Malley, & Ojima, 2009).

## CONFLICT OF INTEREST

None declared.

## AUTHOR CONTRIBUTION

NHC, SAL, and VG developed the research idea and study design. NHC conducted all analyses and drafted the manuscript. SAL and VG provided editorial advice.

## Data Availability

All data and code are available at https://github.com/nhcarter/tiger_abm.
